# An Ontology-based Context-aware System for Smart Homes: E-care@home

**DOI:** 10.3390/s17071586

**Published:** 2017-07-06

**Authors:** Marjan Alirezaie, Jennifer Renoux, Uwe Köckemann, Annica Kristoffersson, Lars Karlsson, Eva Blomqvist, Nicolas Tsiftes, Thiemo Voigt, Amy Loutfi

**Affiliations:** 1 Center for Applied Autonomous Sensor Systems, Örebro University, 70182 Örebro, Sweden; jennifer.renoux@oru.se (J.R.); uwe.kockemann@oru.se (U.K.); annica.kristoffersson@oru.se (A.K.); lars.karlsson@oru.se (L.K.); amy.loutfi@oru.se (A.L.); 2 RISE SICS East, 581 83 Linköping, Sweden; eva.blomqvist@liu.se; 3 RISE SICS, 164 29 Stockholm, Sweden; nicolas.tsiftes@ri.se (N.T.); thiemo.voigt@ri.se (T.V.)

**Keywords:** ambient assisted living, context awareness, Internet of Things, ontologies, activity recognition, smart homes

## Abstract

Smart home environments have a significant potential to provide for long-term monitoring of users with special needs in order to promote the possibility to age at home. Such environments are typically equipped with a number of heterogeneous sensors that monitor both health and environmental parameters. This paper presents a framework called E-care@home, consisting of an IoT infrastructure, which provides information with an unambiguous, shared meaning across IoT devices, end-users, relatives, health and care professionals and organizations. We focus on integrating measurements gathered from heterogeneous sources by using ontologies in order to enable semantic interpretation of events and context awareness. Activities are deduced using an incremental answer set solver for stream reasoning. The paper demonstrates the proposed framework using an instantiation of a smart environment that is able to perform context recognition based on the activities and the events occurring in the home.

## 1. Introduction

A current vision in the area of ICT-supported independent living of elderly people involves populating homes with connected electronic devices (“things”), such as sensors and actuators, and linking them to the Internet. Creating such an Internet of Things (IoT) infrastructure is done with the ambition of providing automated information gathering and processing on top of which e-services for the elderly people (end-users) residing in these homes can be built [1].

As a basis for these services, the information from the home devices can be collected and organized in the form of personal health records (PHRs). In addition to the information contained in the PHRs, the services need to be linked and interfaced to other computerized “things” such as care- and health-related information resources, i.e., electronic health records (EHRs), homemaker services documentation, end-user-generated information and informal caregiver information (e.g., information provided by family, neighbors, social networks, etc.). Semantic interoperability-related technologies play a central role in ambient intelligence systems and their components, such as pervasive/ubiquitous computing, profiling, context awareness and human-centric design. Thus, the IoT infrastructure defines how the things are connected through the Internet and how those things “talk” with other things and communicate with other systems in order to expose their capabilities and functionalities. Technically speaking, advances in the IoT area are mainly supported by continuous progress in wireless sensor/actuator networks’ software applications and by manufacturing low-cost and energy-efficient hardware for device communications. However, the heterogeneity of underlying devices and communication technologies and interoperability in different layers, from communication and seamless integration of devices to interoperability of data/information generated by the IoT resources, are major challenges for expanding generic IoT technologies to efficient ICT-supported services for the elderly people.

In this paper, we present a sensor network in home environments for ambient assisted living that resides within an IoT paradigm, which we call E-care@home. By considering sensor networks in an IoT context, different categories of sensor networks including wearable or environmental sensors can be amalgamated. To provide a context awareness for this IoT setting, a high-level knowledge base is integrated with low-level sensor data. Indeed, much of the literature between middleware and context awareness is highly network-centric. However, in E-care@home, we want to achieve something very different from the previous works in context-aware IoT, and we aim to shift focus from network-centric context awareness to data-centric context awareness. The IoT notion is still very important, as it is the setting for which the E-care@home domain exists. However, what is important in E-care@home is the data, observations and measurements in addition to the node that provides them.

The main contributions of the the paper are two-fold. First, the overall architecture of an IoT setting is presented from the basic sensor infrastructure to the richer levels of data (e.g., the context in the form of objects, events, etc.). Second, we demonstrate how this architecture is used with answer set semantics to identify high level activities that are performed by a user in his/her home. Particular attention in this work is placed on the proposed data model that is able to harmonize information from multiple sources to provide context awareness. We also discuss how the proposed model paves the way towards automatic configuration of the smart home environment.

The remainder of this paper is organized as follows. Section 2 positions E-care@home in relation to the state of the art. An overview of our system is given in terms of the infrastructure and the other technical details in Section 3. Section 4 gives the representational details of our ontological knowledge model, which is followed by a quick introduction of our activity recognition module in Section 5. The experimental results and the explanation of our test bed are also given in Section 6. The paper continues to introduce our next step towards automatic network configuration planning as another application of our knowledge model in Section 7. Finally, we finish the paper with some concluding remarks.

## 2. Related Work

Context awareness in combination with IoT can serve different purposes, such as execution and tagging (annotating) [1]. Tagging is the process of associating context (or meaning) in symbolic form to the sensor data. However, sensor data from one sensor alone may not provide the necessary information to understand a situation. Therefore, tagging may involve fusing together sources of information from other sensors. Execution involves the task of automatically executing services based on the system’s needs. These needs can be highly context dependent, and the services that need to be executed may vary based on the situation at hand.

Both execution and tagging require understanding the sensor data emerging from the E-care@home network. In the area of context-aware computing, this process is achieved via context reasoning, the method to deduct new knowledge based on available information and context [2]. A prerequisite to context reasoning is context modeling, i.e., the creation of relationships between symbolic concepts. The need for reasoning techniques stems from the imperfection of sensor data, i.e., uncertainty, imprecision and erroneous readings [3]. The techniques are evaluated in terms of efficiency, completeness, soundness and interoperability, but few reasoning techniques have been employed in the field of context reasoning. One of the relevant applications of context reasoning in E-care@home is Activity recognition of Daily Living (ADL).

### 2.1. Context-Aware Activity Recognition

Current approaches to reason on human activities are categorized as data-driven and model-driven. Data-driven approaches rely on large volumes of data to learn models for human activities. Examples of this approach include Dynamic Bayesian Networks (DBNs) together with learning methods [4]. Despite the fact that such techniques are highly effective for specific domains, they are brittle with respect to changes in the environment and therefore need retraining when the context changes. There are some works that attempt to remedy this [5], but they still have limitations.

In model-driven approaches, however, activities are defined based on their preconditions rather than learned or inferred from large quantities of data. In particular, sensor data in a mode-driven method are explained based on rules whose preconditions are aligned with changes captured in the data. Examples include [6], where situation calculus is used to specify very rich plans, as well as [7,8,9], all of whom propose rich temporal representations to model the conditions under which patterns of human activities occur. In the literature, there are other model-driven methods contributing to context recognition that are based on ontologies, whose main role is defined in knowledge sharing and reuse [10,11].

Data- and model-driven approaches have complementary strengths and weaknesses. The former provide an effective way to recognize elementary activities from large amounts of continuous data, but rely on the availability of accurately-annotated datasets for training. The latter provide a means to easily customize the system to different operational conditions and users through expressive modeling languages, but is based on the ability of a domain modeler to identify criteria for appropriate recognition from first principles.

However, in E-care@home, context reasoning concerns not only activity recognition. Other events and situations that are of interest to detect involve, e.g., physiological and health-related parameters of the users. Due to the availability of medical or more general health-related knowledge represented in ontologies [12], for E-care@home, we have decided to chose model-driven approach based on ontologies.

The use of context awareness in pervasive computing has previously been studied in the healthcare domain. A number of research projects related to pervasive healthcare and semantic modeling of context have been conducted with the focus on specific aspects, such as health status monitoring, alerts and reminders based on scheduled activities, patient behavior and daily activities’ modeling [13,14]. Many, if not all of these works are highly rule based. For example, the CARAhealthcare architecture [15], which employs fuzzy logic rules, has been shown to enable improved healthcare through the intelligent use of wireless remote monitoring of patient vital signs, supplemented by rich contextual information.

In the following, we describe ontology-based modeling methods and their strengths and weaknesses.

#### 2.1.1. Ontology-Based Context Modeling

More recently, ontologies including knowledge about sensors, their properties and measurements have been proposed to provide a basis to represent the contexts in smart environments. The Semantic Sensor Network (SSN) ontology [16] is an OWL-Description Logic (DL) ontology that allows one to model sensor devices and their capabilities, systems and processes based on earlier models such as SensorMLand O&M.

Structured relationships between concepts are a cornerstone for enabling reasoning based on sensor data. Reasoning over sensor data based on the Semantic Web Rule Language (SWRL) is suggested in [17,18] where rules are applied for different purposes such as implicitly retrieving mentioned knowledge (e.g., events) from sensor data encoded in ontologies (e.g., SSN) [19].

There are other rule-based ontologies proposed in the literature, however with the focus on higher levels of data and not on the sensor level. For instance, [20] proposes an ontology that represents the location of objects for the purpose of activity recognition associated with hybrid statistical and ontological reasoning. The proposed ontology in [21] is also used to perform multilevel activity recognition based on the definition of atomic and more complex human activities given in the ontology. However, the representation of time is implicit or at least not incremental to cover dynamic environments. For instance, in [22], ontological and statistical reasoning are combined to reduce errors in context inference, albeit without addressing temporal relationships between activities.

More specifically, reasoning over rules (e.g., SWRL), which are based on First Order Logic (FOL), is monotonic (https://www.w3.org/Submission/SWRL-FOL/), where adding a new piece of information over time (e.g., new observation) will not change already inferred knowledge. This means that for dynamically-changing environments, it can end up with an inefficient or less precise inference process. It is therefore necessary to study the requirements of various reasoners and their requirements for context modeling to enable other types of reasoning. As stated on p. 432 in [1] “After evaluating several context modeling techniques, it was revealed that incorporating multiple modeling techniques is the best way to produce efficient and effective results, which will mitigate each other’s weaknesses. Therefore, no single modeling technique is ideal to be used in a standalone fashion. There is a strong relationship between context modeling and reasoning”.

Therefore, extending the reasoning beyond the ontological inferences is often necessary since the expressive power of the ontology language is often insufficient for the task at hand. Furthermore, there is lack of (description) logic-based approaches discussed above being capable of dealing with uncertainty and vagueness. Some works approach this by aiming to combine ontological modeling with modeling of uncertainty [23], but fall short in providing a principled approach that preserves the benefits of formal ontologies. Other works suggest a possibly feasible approach, but evaluation is lacking.

E-care@home will contribute towards the process of converting ontological axioms into different parts of a non-monotonic logic program, which is application independent and suitable for reasoning. We will also study alignment techniques that will be able to bridge between heterogeneous ontologies from, e.g., SSN, domain ontologies and personalized user models derived from health records. Our ambition is to investigate and contribute towards methods that will enable interoperability between different contextual models.

Emphasis will be placed on ontological reasoning and enabling the reuse of ontologies with other types of reasoning methods. In particular, we investigate how temporal aspects will be considered explicitly in ontological reasoning. We will consider how to use non-monotonic reasoners (e.g., answer set programming), as well as temporal constraint-based reasoners. We aim to enrich sensor data with annotations that enable a better understanding of what is being measured. To this effect, reasoning will be used not only to describe resources and data at a semantic level, but also to improve analysis and interpretation of sensor data per se. By exploiting relational information between several sensors (e.g., high pulse when high physical activity), we aim to provide context-aware reasoning and annotation of sensor data and to be able to warn in case of anomalies and outliers.

## 3. System Overview

### 3.1. E-care@home Infrastructure

The E-care@home IoT infrastructure consists of three different parts: (1) the E-care@home database; (2) IoT devices; and (3) software and protocols. In order to provide a context-aware interpretation of the heterogeneous sensor data and to provide e-services for the end-users, a smart home ontology has been developed. The following sections provide brief information of the IoT infrastructure and the smart home ontology.

E-care@home adopts Guillemin and Friess’ definition of IoT from 2009 [24], namely: “The Internet of Things allows people and things to be connected Anytime, Anyplace, with Anything and Anyone, ideally using Any path/network and Any service”. The definition is broad, but reflects the breadth of domain applications intended for IoT. Asin and Gascon [25] delimited these domains into twelve categories of which domestic automation and E-health are important for E-care@home.

Prior to the IoT, Sensor Networks (SNs) and related research were used in limited domains and for specific purposes; examples include medical data [26] and health monitoring [27]. Within the IoT context, SNs of different categories can be joined [28]. For E-care@home, this includes body SNs, object SNs and environment SNs. IoT comprises the SN and a thick layer of software, which includes middleware, frameworks and APIs. The software is installed both on computation devices and in the cloud. IoT is concerned with reusing sensors and supporting generic services and functionalities, such as intelligence, semantic interoperability and context awareness, that are also necessary for communication between sensing and actuation [1]. The most essential characteristic of the IoT, where the majority of current research efforts lie, is the requirement of a middleware solution. “Middleware is a software layer that stands between the networked operating system and the application and provides well known reusable solutions to frequently encountered problems like heterogeneity, interoperability, security, dependability” ([29], p. 244). There are many works describing the basic functionalities of a middleware, e.g., [30,31,32]. One major research gap in IoT is in providing middleware with context awareness [1,33], and the current intersection between middleware and context awareness in the literature is network-centric. For example, CASNuses the low-level context, e.g., the remaining energy of a node, the location and orientation of a sensor to decide energy-efficient routing [1].

### 3.2. E-care@home Database

Data collection and processing in E-care@home are centered around a database. This allows one to decouple data processing from heterogeneous data sources (e.g., different sensors/networks, different software for data processing and filtering). In addition, the database also collects annotations from various sources, such as activity labels added by users or context inferred by reasoners. Apart from the technical benefits, this solution also promotes publishing smart home datasets that are currently hard to find.

### 3.3. IoT Devices

As mentioned above, we include data from a variety of sources. In an existing smart home laboratory at Örebro University, we have a network of sensor nodes using XBee modules (https://www.digi.com/products/xbee-rf-solutions) for wireless communication. These sensor nodes are used for different measurement purposes (light, motion, temperature, contact, pressure). XBee nodes are relatively easy to setup, but do not allow for reconfiguration during runtime.

We also use a network of IoT devices based on the Contiki operating system. These nodes (Zolertia RE-Motes (http://zolertia.io/product/hardware/re-mote)) support custom software and, through Contiki, support a variety of protocols (see the next section). This will allow us to update sensor configurations during runtime based on the current context and the possibility to adjust the behavior of communication protocols based on the requirements of the configuration planner. Finally, we employ Shimmer wearable sensors (http://www.shimmersensing.com/) to collect medical data including heart rate, respiration and blood pressure about the user.

### 3.4. Software and Protocols

**Figure 1:**
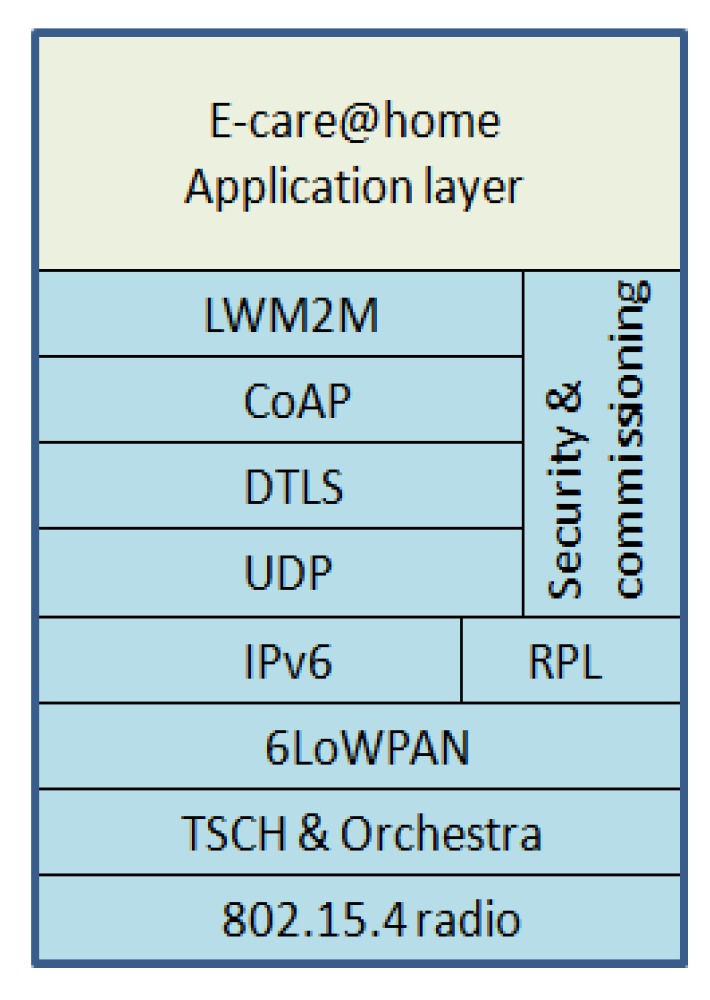
The secure, low-power communication stack that we use in Contiki-based nodes for environmental sensing within E-care@home deployments. LWM2M, Lightweight Machine to Machine protocol; TSCH, Time-Slotted Channel Hopping protocol.

The E-care@home IoT infrastructure makes extensive use of state of the art communication protocols that have been implemented in the open-source Contiki operating system [34]. A key requirement for E-care@home deployments in smart homes is that the IoT devices must communicate in a robust, yet energy-efficient manner to ensure dependable operation. Towards this end, we have enhanced the low-power IPv6 communication stack in Contiki with new implementations of low-power wireless protocols and augmentations of existing protocol implementations. Figure 1 shows the key components of this communication stack.

We have implemented the Time-Slotted Channel Hopping protocol (TSCH) for Contiki and developed a novel time slot scheduler named Orchestra, which allows nodes to set up efficient schedules based on the local networking state [35]. Compared with the state-of-the-art ContikiMAC protocol used in Contiki, TSCH with Orchestra can enhance packet reception rates and reduce radio duty cycles by setting up dedicated time slots over multiple channels for different types of traffic and neighbor nodes. We have further contributed open-source mechanisms for Contiki’s RPLrouting protocol implementation [36] that improve the robustness of two-way communication within IoT networks.

At the application layer, we use standard UDP, the Constrained Application Protocol (CoAP) [37] and the Open Mobile Alliance’s Lightweight Machine to Machine protocol (LWM2M) [38] to support flexible reporting of sensor samples and reconfiguration of devices using standard protocols. These protocols have been designed to support a variety of use cases and traffic patterns, while having relatively low requirements on implementation complexity and communication overhead. Additionally, LWM2M supports simple integration of end-to-end encryption with DTLS, which is a particularly beneficial quality for mission-critical systems in the e-health domain.

## 4. SmartHome Ontology

In this section, we outline the details of our ontological knowledge model called the SmartHomeontology, which is proposed to assist the development process of sensorized environments. The SmartHome ontology is described in terms of its modules representing different aspects including physical (e.g., the structure of the environment, network setting or entities in the environment) and conceptual (e.g., events or observation processes) aspects of a smart environment. This ontology also allows us to to describe the capabilities of the various sensors (and actuators) in the environment, as well as capturing the interpretations of the sensor data. Attempting to capture all of the required dependencies in a single ontology would result in a large and complex model that is difficult to maintain over time, e.g., when sensors/devices are added to or removed from the environment or when the monitoring requirements of the assisted person change. Additionally, when using ontologies in a system that requires near real-time reasoning and reactions by the system, the reasoning complexity is an essential parameter of the ontologies. For these reasons, we propose the use of a network of ontology modules, which could be considered as ontology patterns [39], instead of merely directly using a monolithic ontology. In order to create such a network of modules, we have applied an incremental methodology, starting by creating and linking small (in size) modules, which are mainly aligned to and extend from the Semantic Sensor Network Ontology (SSN) [16] based on the upper ontology DUL (DOLCE Ultra Light: www.ontologydesignpatterns.org/ont/dul/DUL.owl) providing the semantic foundation. Doing so helps us to realize the main links in the eventual ontology and consequently makes a stable and at the same time flexible network of concepts that can be updated with the minimum change propagation on the entire ontology.

**Figure 2:**
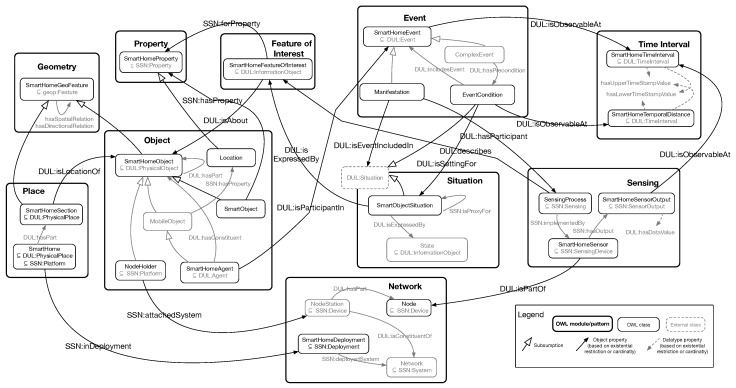
Overview of the SmartHome ontology composed of 10 ontology modules linked together. SSN, Semantic Sensor Network; DUL, DOLCE Ultra Light.

Figure 2 represents an abstract overview of the SmartHome ontology (http://ecareathome-ontology.mpi.aass.oru.se/SmartHome.owl) in terms of its modules and their relations. The figure is somewhat simplified in order to be more readable, e.g., most inverse relations have been omitted, as well as some of the alignments to external classes and properties. The ontology is composed of 10 ontology modules that each represents a single principal feature of a smart home. As we will see in the following subsections, most of the ontology modules are extending SSN (which in turn relies on DUL). The names of the concepts and relations taken from the aforementioned ontologies are represented in the form of hyper-links, referring to the OWL definition of the concepts. In the following sections, we briefly introduce the ontology modules (patterns) in the SmartHome ontology. Since the ontology modules are represented in Description Logic (DL) language, we first briefly explain the notations used in the following sections (for further details, we refer the readers unfamiliar with the DL syntax to the Basic DL tutorial in [40]):

- The expression “**name1** : **name2**” refers to the entity **name2**, which belongs to the ontology **name1**.
- The **subsumption**relation shown as “A⊑B” means concept **A** is a subset (a specialization) of concept **B**.
- The **full existential quantification** shown as “∃R.C” indicates all of the concepts whose instances have at least one relation with concept **C** via property **R**.
- The **number restriction** shown as “= n **R**”, where n is a numerical value, indicates an ontological concept whose instances are related to other concepts/values n-times via the property **R**.

### 4.1. SmartHome_TimeInterval Pattern

The pattern **SmartHome_TimeInterval**represents time intervals between any two timestamps through an observation process. This pattern is used in different occasions, such as in capturing an event occurring within a specific time interval (e.g., *TV was on between 11:00 a.m. and 11:30 a.m.*) or in recognizing events whose preconditions need to be captured within a specific time distance regardless of the real date/time at which they occur (e.g., *eating lunch usually starts between 1 and 30 min after a cooking process ends*). The two main classes of this pattern are *SmartHomeTimeInterval* (represents an interval between two specific time-date values) and *SmartHomeTemporalDistance* (represents an interval between two timestamps), which are subsumed by the class DUL:TimeInterval. These classes indicate a boundary for the time interval using the two properties *hasLowerTimeStampValue* and *hasUpperTimeStampValue*.

(1) $$\begin{array}{cc} SmartHomeTimeInterval⊑\; & \mathrm{DUL}:\mathrm{TimeInterval}\;⊓\\ & \;=1\;hasLowerTimeStampValue.(\mathrm{xsd}:\mathrm{dateTime})\\ & \;=1\;hasUpperTimeStampValue.(\mathrm{xsd}:\mathrm{dateTime}) \end{array}$$

(2) $$\begin{array}{cc} SmartHomeTemporalDistance⊑\; & \mathrm{DUL}:\mathrm{TimeInterval}\;⊓\\ & \;=1\;hasLowerTimeStampValue.(\mathrm{xsd}:\mathrm{long})\\ & \;=1\;hasUpperTimeStampValue.(\mathrm{xsd}:\mathrm{long}) \end{array}$$

### 4.2. SmartHome_Geometry Pattern

Apart from the temporal aspect of a smart environment, the representational model needs to also cover the spatial aspects of entities (e.g., objects, rooms, etc.) occupying space. For this, we have extended the GeoSPARQL (The OGC (Open GeoSpatial Consortium) GeoSPARQL standard defines an adequate vocabulary for representing geospatial data enabling qualitative spatial reasoning based on geometrical computations) ontology [41] in order to provide a basis for the representation of the geometry of entities, as well as the topological and directional relations between any pair of geometrical objects. There are many situations where we need to (symbolically) localize objects with respect to the position/location of the other objects (e.g., *the kitchen is connected to the living-room*, *living room is at the right side of the bedroom* or *the bed is close to the left wall*).

The main class of this pattern is *SmartHomeGeoFeature* subsumed by the class geop:Feature (the acronym *geop* refers to the ontology GeoSPARQL). According to GeoSPARQL, a feature represents a spatial object that has a geometry. We further specialize this definition by saying that each feature is also in a spatial and a directional relation with at least one other feature:

(3) $$\begin{array}{cc} SmartHomeGeoFeature⊑\; & \mathrm{geop}:\mathrm{Feature}\;⊓\\ & \;\exists \;\mathrm{geop}:\mathrm{hasGeometry}.\mathrm{geop}:\mathrm{Geometry}\\ & \;\exists \;hasSpatialRelation.SmartHomeGeoFeature\\ & \;\exists \;hasDirectionalRelation.SmartHomeGeoFeature \end{array}$$

### 4.3. SmartHome_Property Pattern

By property, we refer to a feature of a physical object that is the interest of an observation process in a smart environment. These properties vary depending on the category of objects (e.g., mobile or non-mobile objects) and also the measurement criteria (e.g., location of objects) based on which the observation process is conducted. The pattern SmartHome_Property is used to more tightly couple the representation of physical objects with some features or properties, which are measurable by sensors.

We assume that objects in the context of smart environments are represented as the subclasses of the class DUL:PhysicalObject (see Section 4.4). Under this assumption, a property of an object indicates a feature of the object (e.g., the *pressure* of the couch, the *location* of the chair or the *luminosity* of the TV) and is defined within the class SSN:Property.

(4) $$\begin{array}{cc} SmartHomeProperty⊑\; & \mathrm{SSN}:\mathrm{Property}\;⊓\\ & \;\exists \;\mathrm{SSN}:\mathrm{isPropertyOf}.SmartObject \end{array}$$

### 4.4. SmartHome_Object Pattern

The pattern **SmartHome_Object** allows us to define objects based on their features in the context of smart environments. The class DUL:PhysicalObject provides a suitable representational basis for the objects’ taxonomy. The class *SmartHomeObject* is categorized into two types of *SmartObject* and *NodeHolder*. By *SmartObject*, we refer to those objects that are the interest of an observation process.

In order to also be able to reflect the spatial relations between objects (e.g., the “fridge is connected to the cabinet”) or between an object and a place (see Section 4.9) where it is located (e.g., “the bed is located at the left side of the bedroom”), it is required to define the *SmartHomeObject* also as a *SmartHomeGeoFeature* given in the pattern **SmartHome_Geometry**:

(5) $$\begin{array}{cc} SmartHomeObject⊑\; & \mathrm{DUL}:\mathrm{PhysicalObject}\;⊓\\ & \;SmartHomeGeoFeature\;⊓\\ & \;\exists \;\mathrm{DUL}:\mathrm{hasLocation}.SmartHomeSection \end{array}$$

(6) $$\begin{array}{cc} SmartObject⊑\; & SmartHomeObject\;⊓\\ & \;\exists \;\mathrm{SSN}:\mathrm{hasProperty}.SmartHomeProperty\; \end{array}$$

A *NodeHolder* is also a *SmartHomeObject* representing objects that are not necessarily monitored by sensors, however acting as a platform (or holder) for a device (a node) in a sensor network to ease the process of sensor installation (see Section 4.8). This separation between smart objects (which are the interest of the observation process) and node holders provided by this pattern is useful for the sensor localization process particularly used by a configuration planner; one of whose tasks will be to check the status/functionality of sensors by using other sensors (e.g., mobile robots).

(7) $$\begin{array}{cc} NodeHolder⊑\; & SmartHomeObject\;⊓\\ & \;\mathrm{SSN}:\mathrm{Platform}\;⊓\\ & \;\exists \;\mathrm{SSN}:\mathrm{attachedSystem}.NodeStation \end{array}$$

Another categorization of smart objects that has been considered in the object pattern is about their mobility. An objects is considered as mobile only if its location as one of its properties (*Location* ⊑ *SmartHomeProperty*) can change.

(8) $$\begin{array}{cc} MobileObject⊑\; & SmartHomeObject\;⊓\\ & \;\exists \;\mathrm{SSN}:\mathrm{hasProperty}.Location\; \end{array}$$

The class *MobileObject* can be further specialized and form another type of object that is able to be proactive and participate in some events. The class *SmartHomeAgent* subsumed by the class DUL:Agent allows us to represent smart home objects, such as inhabitants including persons or pets, that can be involved in an activity or an event (e.g., a cat at home is defined as an agent as it can to be involved in activities such as “*sitting on the couch*” or, likewise, a person is also an agent at home as he/she is often involved in various activities, such as “*watching TV*”, etc.). Each smart home agent can own, as its constituent, a *SmartHomeObject* considered in a sensing process. A good example of such constituents is comprised of the body parts of the agent, which as a *SmartObject* (e.g., the *heart* of a person) is monitored by sensors. A constituent of a smart home agent, in general, is referring to objects whose locations are the same as the location of the agent. More specifically, a constituent can express other objects physically attached to the agent. However, a constituent does not need to be permanently attached to the agent. For instance, a chair might be deemed as a constituent as long as it is held by the agent.

$$\begin{array}{cc} SmartHomeAgent⊑\; & \mathrm{DUL}:\mathrm{Agent}\;⊓\;MobileObject\;⊓\\ & \;\exists \;\mathrm{DUL}:\mathrm{hasConstituent}.SmartHomeObject\;⊓\\ & \;\exists \;\mathrm{DUL}:\mathrm{isParticipantIn}.\mathrm{DUL}:\mathrm{Event} \end{array}$$

### 4.5. SmartHome_FeatureOfInterest Pattern

A feature of interest commonly refers to a concept deemed as the interest of an observation process (e.g., *temperature of the oven*). There are many situations where a feature of interest is seen only as a piece of information that indicates both the object (e.g., *oven*) and its specific property (e.g., *temperature*), without mentioning its numerical values (e.g., $70{\;}^{\circ }C$) or even symbolic states (e.g., *warm*, *cold*). Using the class DUL:InformationObject (according to DUL, an information object is defined as a piece of information independent from how it is concretely realized), the pattern SmartHome_FeatureOfInterest allows us to define a feature of interest regardless of its measurement details or the possible states that it might have.

(9) $$\begin{array}{cc} SmartHomeFeatureOfInterest⊑\; & \mathrm{DUL}:\mathrm{InformationObject}\;⊓\\ & \;\exists \;\mathrm{DUL}:\mathrm{isAbout}.SmartObject\;⊓\\ & \;\exists \;\mathrm{SSN}:\mathrm{forProperty}.SmartHomeProperty \end{array}$$

### 4.6. SmartHome_Situation Pattern

A feature of interest can be declaratively expressed to indicate a specific situation. More specifically, a situation refers to a particular *state* of a *feature of interest* (e.g., *the temperature of the living room is warm*). Although these states are usually time dependent, in the SmartHome ontology, we have considered an abstract and time independent representation for the situation concept. This representation can be later augmented with time in other patterns, such as event-related patterns associated with temporal properties (see Section 4.10).

(10) $$\begin{array}{cc} SmartObjectSituation⊑\; & \mathrm{DUL}:\mathrm{Situation}\;⊓\\ & \;\exists \;\mathrm{DUL}:\mathrm{isExpressedBy}.SmartHomeFeatureOfInterest\;⊓\\ & \;\exists \;\mathrm{DUL}:\mathrm{isExpressedBy}.State\; \end{array}$$

(11) $$\begin{array}{cc} State⊑\; & \mathrm{DUL}:\mathrm{InformationObject}\;⊓\\ & \;\exists \;\mathrm{DUL}:\mathrm{expresses}.SmartObjectSituation\; \end{array}$$

In the above definition, we see the class ***State***, whose individuals are assumed to declaratively express a feature of interest regardless of how this expression is realized.

### 4.7. SmartHome_Sensing Pattern

A sensing process is defined as the process of monitoring a feature of interest using a sensing device. This process is seen as a method (by method, we refer to the class DUL:Method ⊑SSN:Process⊑ SSN:Sensing), which monitors a phenomenon (or a feature of interest) in order to describe its state at different time points. A sensing process furthermore depends on a sensing device whose outputs are regarded as the result of the process. As shown in Figure 2, this pattern is connected to the pattern **SmartHome_FeatureOfInterest** and also **SmartHome_Network** (see Section 4.8), which is responsible for the physical implementation of a sensing process.

(12) $$\begin{array}{cc} SensingProcess⊑\; & \mathrm{SSN}:\mathrm{Sensing}\;⊓\\ & \;\exists \;\mathrm{SSN}:\mathrm{implementedBy}.SmartHomeSensor\;⊓\\ & \;\exists \;\mathrm{DUL}:\mathrm{describes}.SmartHomeFeatureOfInterest\; \end{array}$$

### 4.8. SmartHome_Network Pattern

A network in a smart environment is defined as a system containing different types of devices such as nodes and node stations. By node, we mean a communication module that indicates either a sending or a receiving data module in a network. Each node, depending on its type, can be a part of a node station representing another type of device that contributes to establishing a network. Each node station contains a node along with other things, including a sensor, power supplies, batteries, etc.

The whole process of a network setup regardless of its exact technical details is represented by a non-physical concept called deployment. The pattern **SmartHome_Network** unifies the representation of home automation installations, a variety of which can be found in different systems.

The class **SmartHomeDeployment** extends the class SSN:Deployment and indicates a platform as a physical place (e.g., a smart home; see Section 4.9) where a system (e.g., a network) is deployed (SSN:deployedOnPlatform is the inverse property of SSN:inDeployment).

(13) $$\begin{array}{cc} SmartHomeDeployment⊑\; & \mathrm{SSN}:\mathrm{Deployment}\;⊓\\ & \;\exists \;\mathrm{SSN}:\mathrm{deployedOnPlatform}.SmartHome\;⊓\\ & \;\exists \;\mathrm{SSN}:\mathrm{deployedSystem}.Network \end{array}$$

The class **Network** is also subsumed by the class SSN:System. This class is further specialized by relating it to a deployment process, as well as to its constituents:

(14) $$\begin{array}{cc} Network⊑\; & \mathrm{SSN}:\mathrm{System}\;⊓\\ & \;\exists \;\mathrm{DUL}:\mathrm{hasConstituent}.NodeStation\;⊓\\ & \;\exists \;\mathrm{SSN}:\mathrm{hasDeployment}.SmartHomeDeployment \end{array}$$

**NodeStation** represents a SSN:Device (either a *SenderNodeStation* or a *ReceiverNodeStation*), which is located on a platform (e.g., a node holder) in the environment and can contain a number of nodes (SSN:onPlatform is the inverse property of SSN:attachedSystem).

(15) $$\begin{array}{cc} NodeStation⊑\; & \mathrm{SSN}:\mathrm{Device}\;⊓\\ & \;\exists \;\mathrm{DUL}:\mathrm{hasPart}.Node\;⊓\\ & \;\exists \;\mathrm{DUL}:\mathrm{isConstituent}.Network\;⊓\\ & \;\exists \;\mathrm{SSN}:\mathrm{onPlatform}.NodeHolder \end{array}$$

Moreover, the class **Node** represents a SSN:Device, either a *DataReceiverNode* or a *DataSenderNode* contained in a node station:

(16) $$\begin{array}{cc} Node⊑\; & \mathrm{SSN}:\mathrm{Device}\;⊓\\ & \;\exists \;\mathrm{DUL}:\mathrm{isPartOf}.NodeStation \end{array}$$

### 4.9. SmartHome_Place Pattern

The meaning of a place in the context of smart home is two-fold. First, by a place, we mean the entire smart environment, which as a platform holds the deployment of a sensor network and may also be composed of several sections.

(17) $$\begin{array}{cc} SmartHome⊑\; & \mathrm{DUL}:\mathrm{PhysicalPlace}\;⊓\\ & \;\mathrm{SSN}:\mathrm{Platform}\;⊓\\ & \;\exists \;\mathrm{SSN}:\mathrm{inDeployment}.SmartHomeDeployment\;⊓\\ & \;\exists \;\mathrm{DUL}:\mathrm{hasPart}.SmartHomeSection \end{array}$$

The second meaning of a place refers to each section of the main place with a specific identity that can accommodate different objects. Each section in a smart home also has a geometry and, therefore, can be in spatial relation with the other sections.

(18) $$\begin{array}{cc} SmartHomeSection⊑\; & \mathrm{DUL}:\mathrm{PhysicalPlace}\;⊓\\ & \;SmartHomeGeoFeature\;⊓\\ & \;\exists \;\mathrm{DUL}:\mathrm{isLocationOf}.SmartHomeObject\;⊓\\ & \;\exists \;\mathrm{DUL}:\mathrm{isPartOf}.SmartHome \end{array}$$

### 4.10. SmartHome_Event Pattern

From the observation process viewpoint, an event can be defined as either a manifestation or a complex event, which occurs within a time interval (represented via the class **SmartHomeTemporalDistance** explained in Section 4.1) or at a specific time point (where lower and the upper bound of the interval are equal) and involves at least one agent.

(19) $$\begin{array}{cc} SmartHomeEvent⊑\; & \mathrm{DUL}:\mathrm{Event}\;⊓\\ & \;\exists \;\mathrm{DUL}:\mathrm{hasParticipant}.SmartHomeAgent\;⊓\\ & \;\exists \;\mathrm{DUL}:\mathrm{isObservableAt}.SmartHomeTimeInterval \end{array}$$

Manifestation is a specific **SmartHomeEvent** that is directly captured from sensor data and represents the occurrence of a situation (i.e., the *SmartObjectSituation* class defined in Section 4.6) through a sensing process.

(20) $$\begin{array}{cc} Manifestation⊑\; & SmartHomeEvent\;⊓\\ & \;\exists \;\mathrm{DUL}:\mathrm{isEventIncludedIn}.SmartObjectSituation \end{array}$$

**Figure 3:**
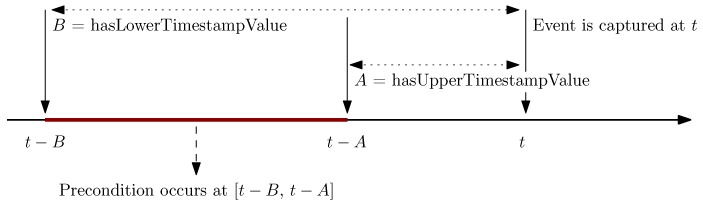
Temporal relation between an event occurs at time tand its precondition expected to be identified during the time interval: [t-A .. t-B].

However, a complex event, as its name indicates, represents an event whose occurrence depends on a number of preconditions [42]. Each precondition as such represents a specific situation assumed to be observed within an interval (or time point) with a specific temporal distance from the time point at which the event occurs (see Figure 3).

(21) $$\begin{array}{cc} ComplexEvent⊑\; & SmartHomeEvent\;⊓\\ & \;\exists \;\mathrm{DUL}:\mathrm{hasPrecondition}.EventCondition \end{array}$$

The class ***EventCondition*** as a DUL:Situation represents preconditions of a complex event (in the form of a situation or, more specifically, a *SmartObjectSituation*) needed to be captured within a specific temporal distance (represented by the class **SmartHomeTemporalDistance** explained in Section 4.1) from the timestamp of the complex event (see Figure 3).

(22) $$\begin{array}{cc} EventCondition⊑\; & \mathrm{DUL}:\mathrm{Situation}\;⊓\\ & \;\exists \;\mathrm{DUL}:\mathrm{isPreconditionOf}.ComplexEvent\;⊓\\ & \;\exists \;\mathrm{DUL}:\mathrm{isObservableAt}.SmartHomeTemporalDistance\;⊓\\ & \;\exists \;\mathrm{DUL}:\mathrm{isSettingFor}.SmartObjectSituation \end{array}$$

**Figure 4:**
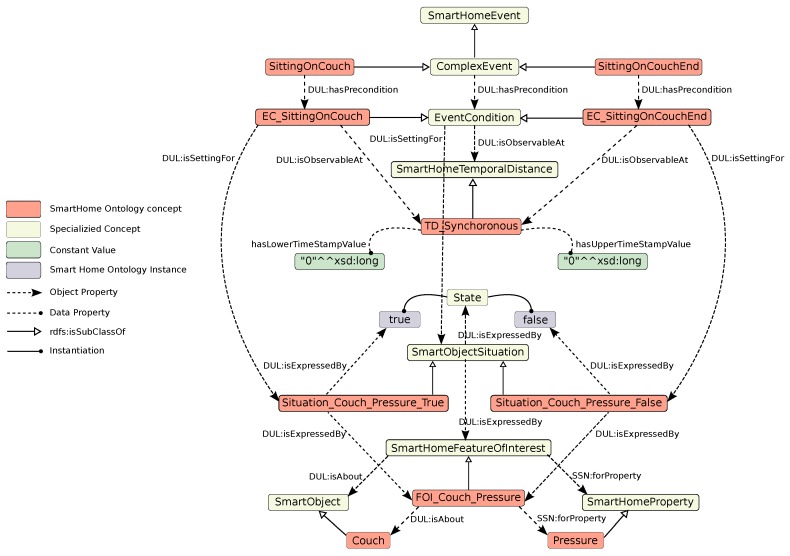
Representation of the two complex events **SittingOnCouch** and **SittingOnCouchEnd** in the SmartHome ontology.

As an example, the DL definitions of the complex event *sittingOnCouch* and its ending *sittingOnCouchEnd* are given in the following. For further clarification, we have first provide the ontological definitions of their dependencies including the smart objects, feature of interests, etc. This representation has also been visualized in Figure 4.

(23) $$\begin{array}{ccc} Couch & ⊑\; & SmartObject \end{array}$$

(24) $$\begin{array}{ccc} Pressure & ⊑\; & SmartHomeProperty \end{array}$$

(25) $$\begin{array}{ccc} FOI\_Couch\_Pressure & ⊑\; & SmartHomeFeatureOfInterest\;⊓\;\exists \;\mathrm{DUL}:\mathrm{isAbout}.Couch\;⊓\;\exists \;\mathrm{SSN}:\mathrm{forProperty}.Pressure \end{array}$$

(26) $$\begin{array}{ccc} Situation\_Couch\_Pressure\_True & ⊑\; & SmartObjectSituation\;⊓\;\exists \;\mathrm{DUL}:\mathrm{isExpressedBy}.FOI\_Couch\_Pressure\;⊓\;\exists \;\mathrm{DUL}:\mathrm{isExpressedBy}.true \end{array}$$

(27) $$\begin{array}{ccc} Situation\_Couch\_Pressure\_False & ⊑\; & SmartObjectSituation\;⊓\;\exists \;\mathrm{DUL}:\mathrm{isExpressedBy}.FOI\_Couch\_Pressure\;⊓\;\exists \;\mathrm{DUL}:\mathrm{isExpressedBy}.false \end{array}$$

(28) $$\begin{array}{ccc} TD\_Synchronous & ⊑\; & SmartHomeTemporalDistance\;⊓\;(=1\;hasLowerTimeStampValue.0)\;⊓\;(=1\;hasUpperTimeStampValue.0) \end{array}$$

(29) $$\begin{array}{ccc} SittingOnCouch & ⊑\; & ComplexEvent\;⊓\;\exists \;\mathrm{DUL}:\mathrm{hasPrecondition}.EC\_SittingOnCouch \end{array}$$

(30) $$\begin{array}{ccc} EC\_SittingOnCouch & ⊑\; & EventCondition\;⊓\;\exists \;\mathrm{DUL}:\mathrm{isObservableAt}.TD\_Synchronous\;⊓ \end{array}$$

(31) $$\begin{array}{c} \;\exists \;\mathrm{DUL}:\mathrm{isSettingFor}.Situation\_Couch\_Pressure\_True \end{array}$$

(32) $$\begin{array}{ccc} SittingOnCouchEnd & ⊑\; & ComplexEvent\;⊓\;\exists \;\mathrm{DUL}:\mathrm{hasPrecondition}.EC\_SittingOnCouchEnd \end{array}$$

(33) $$\begin{array}{ccc} EC\_SittingOnCouchEnd & ⊑\; & EventCondition\;⊓\;\exists \;\mathrm{DUL}:\mathrm{isObservableAt}.TD\_Synchronous\;⊓ \end{array}$$

(34) $$\begin{array}{c} \;\exists \;\mathrm{DUL}:\mathrm{isSettingFor}.Situation\_Couch\_Pressure\_False \end{array}$$

## 5. Context-Aware Reasoning for Activity Recognition

One important task of our system, which is also typical of similar types of systems having been frequently reported in the literature, is to identify the activities of a person in the environment such as “sleeping”, “watching TV”, “cooking”, etc. In addition to being able to identify an activity, we are also interested in the time and location of where the activity is carried out. By specializing the ontological classes in the modules of the SmartHome ontology, we can provide the representational basis for the context inference process. However, in order to continuously capture changes (either in the form of activities or other events) in the environment, a stream reasoning process is required. By stream reasoning, we mean a logical reasoning process applied upon data streams in real time to be able to recognize the current situation of the environment [43].

Since context awareness is assumed to be in real time, the stream reasoning process should be stable, as well as efficient, specifically in dealing with unknown situations that are likely to happen in a smart environment due to either the lack of observations or sensor failures. In many context awareness applications, the reasoning process is deductive, which relies on monotonic logic and specify conditions under which events may occur. However, in order to keep the performance of the reasoner suitable for real-time monitoring, deductive models usually provide less declarative language than models relying on non-monotonic logic. Non-monotonic reasoning is based on belief revision, which allows a new hypothesis or observation to contradict the previously-inferred beliefs. Due to the inherent uncertainty of sensor data, hypotheses in the form of sensor observations are not always complete. In particular, interpretation of sensor data about the environment evolves over time. Since non-monotonic reasoning is known to be efficient in dealing with incomplete data, in this work, we have applied answer set logic as a non-monotonic logic for sensor data [44]. Answer Set Programming (ASP) is defined as a declarative paradigm based on the stable model semantics (or answer sets), according to which the domain of discourse can be modeled based on the Closed World Assumption (CWA). According to its CWA, the stable model semantics can model situations where by default, something (or the lack of something) is false (or true).

**Figure 5:**
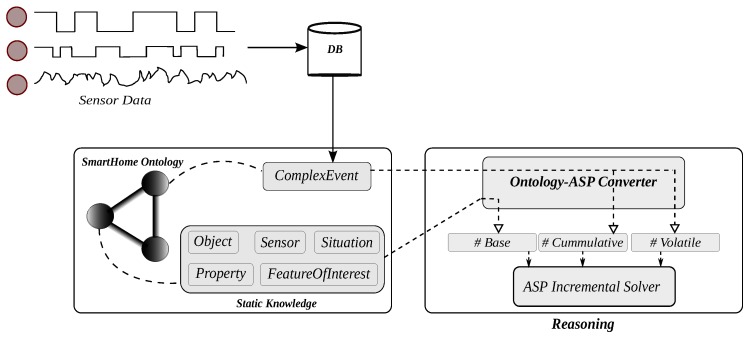
Context inference system architecture from sensing to reasoning. ASP, Answer Set Programming.

Figure 5 illustrates the architecture of the system composed of two main components: the ontological knowledge model and the reasoner. The knowledge represented in the ontology is divided into two types of static and dynamic knowledge. The static knowledge, which is time-independent, is also referred to as the background knowledge and modeled by time-independent patterns in the ontology, such as **SmartHome_Object**, **SmartHome_Property**, **SmartHome_Place**, etc. On the contrary, the dynamic knowledge refers to time-dependent knowledge modeled by patterns, such as **SmartHome_Event** or **SmartHome_Sensing**, whose definitions rely on the **SmartHome_TimeInterval** pattern.

The idea behind the context inference process in our system is converting the ontological knowledge and the data streams, which are, as shown in Figure 5, first recorded in the database, into logic programs solvable by the ASP solver. A non-monotonic logic program *P* consists of a set of rules $r_{i}$ of the form:

(35) $$r_{i}:a_{0}\leftarrow a_{1},\cdots ,a_{m},not\;a_{m+1},\cdots ,not\;a_{n}$$

where for m,n≥0, $a_{i}$ represents a logical predicate that can either represent static or dynamic knowledge. For instance, *chair(object1)*, regardless of time, defines the specific object *object1* as a chair (where Chair⊑SmartObject) in the ontology. Likewise, the time-dependent predicate *event(object1, pressure, true, t)* indicates a change in the environment at time *t* when the *pressure* sensor attached to the chair *object1* was activated.

The ASP solver used in this work is the incremental solver called Clingo [45]. Clingo is used to solve any logic program R, which is composed of three different logic programs, namely the base (B), cumulative (P[t]) and volatile (Q[t]) logic programs. The base program B refers to a time independent logic program, which only represents the static knowledge. On the other hand, the two logic programs P and Q indicate dynamic knowledge that can change over the time. In order to model the temporal behavior of the dynamic phenomena, these two dynamic logic programs are equipped with a parameter *t* showing the current timestamp. The size of the program P, which represents the cumulative knowledge, increases with the growth of *t*. However, the content of Q always indicates the situation at the current timestamp. In other words, by changing the timestamp, the contents of Q are overwritten with new content. Assuming that the timestamp has reached its *j*-th step, the content of the incrementally-extended program R at the step *j* is formally shown in the following:

(36) $$R\left[t_{j}\right]=B\cup \left(\overset{j}{\underset{i=1}{⋃}}P\left[t_{i}\right]\right)\cup Q\left[t_{j}\right]$$

The eventual answer sets of R[t] will be considered as the solution for the logically-modeled problem statement, which in our case is providing an explanation for the current situation in terms of the inferred events and activities.

As illustrated in Figure 5, the contents of the ontology are translated into logic programs solvable by the incremental ASP solver. The definition of instances belonging to the time-independent concepts in the ontology forms the basic logic program (B). These concepts include objects (e.g., *chair(object1)*, *couch(object2)*, *tv(object3)*, etc.) and the features of interest that express the relations between an object and its property, which is the interest of the observation process (e.g., *foi(object1, pressure)*, *foi(object3, luminosity)*, etc.). Furthermore, the definition (i.e., preconditions) of events given in the ontology as the time-dependent knowledge is also translated into the cumulative (P) logic program. Once a change (an event) is detected in a signal, the volatile logic program (Q) triggers the solver in order to infer at least one explanation for this event.

The dynamic knowledge, as explained above, is mainly represented by the **SmartHome_Event** pattern. As shown in the example given in Section 4.10, what we exactly explain in this pattern is the set of preconditions required to infer both the starting and ending of each complex event.

Given the axioms in the ontology about the context including objects, features of interest, situations, etc., the logic program generators will generate three different rules for each complex event. The first rule indicates the conditions under which the system can infer the starting of the complex event. For instance, the three rules related to the complex event *sittingOnCouch* shown in Figure 4 are generated as follows:

(37) $$\begin{array}{c} \mathrm{starting}\;\mathrm{rule}:\\ \;complexEvent(sittingOnCouch,\;t)\;:-\\ \;situation(couch,pressure,true,t),\;not\;complexEvent(sittingOnCouch,\;t-1). \end{array}$$

(38) $$\begin{array}{c} \mathrm{ending}\;\mathrm{rule}:\\ \;complexEvent(sittingOnCouchEnd,\;t)\;:-\\ \;situation(couch,pressure,false,t),\;\;complexEvent(sittingOnCouch,\;t-1). \end{array}$$

(39) $$\begin{array}{c} \mathrm{continuation}\;\mathrm{rule}:\\ \;complexEvent(sittingOnCouch,\;t)\;:-\\ \;complexEvent(sittingOnCouch,\;t-1),\;not\;complexEvent(sittingOnCouchEnd,\;t). \end{array}$$

The starting rule says that we infer *sittingOnCouch* at time t if at this time the pressure sensor on the couch has been activated, and at the previous time stamp (t-1), there was no instance of *sittingOnCouch* inferred. Likewise, the ending rule indicates that *sittingOnCouchEnd* is inferred at time *t* if at this time, the pressure sensor on the couch is not active, and we have been sitting on the couch at the previous time stamp (t-1). Using the similar negation operator in the continuation rule, we can infer the truth of the *sittingOnCouch* at each time stamp until its ending predicate is inferred.

## 6. SmartHome Deployment

The E-care@home system has been implemented and tested in a living lab environment. This section presents the configuration of the smart environment, as well as the whole process from the data recording to the activity recognition.

**Figure 6:**
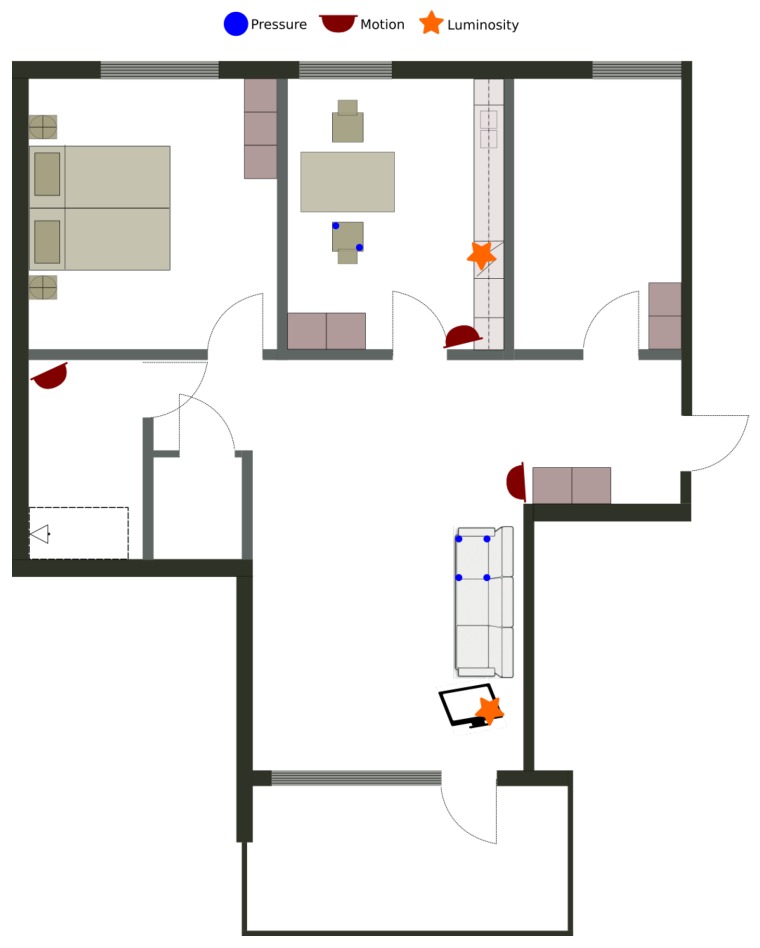
Map of the smart apartment with the installed sensors.

The aforementioned smart environment is a 84 m^2^ apartment including an entrance, a living room, a kitchen, a bedroom and a bathroom. For this deployment, only the living room, the kitchen and the bathroom have been sensorized. Figure 6 presents the map of the apartment with the location of the sensors.

**Figure 7:**
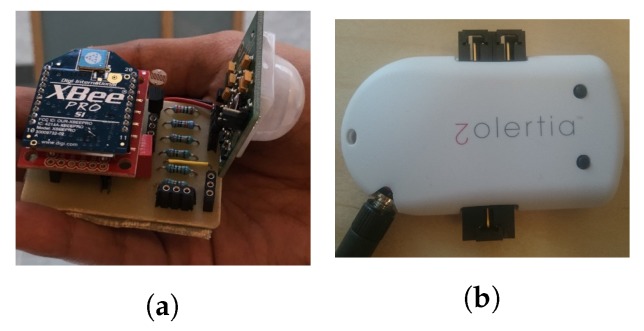
The sensors. (**a**) An X-bee node with a motion sensor; (**b**) a zolertia node with a light sensor.

The sensors used are connected to XBee nodes and Zolertia nodes, as presented in Section 3.3 (see Figure 7). The XBee nodes are used for motion sensors (one per room) and pressure sensors (four under the couch and two under the chair). The Zolertia nodes are used for luminosity sensors to monitor the state of the television and the state of the oven (on/off).

Sensor data are sent to a computer through XBee and Zolertia receiver nodes. This computer continuously receives data coming from different sensors and stores them in the E-care@home database.

**Figure 8:**
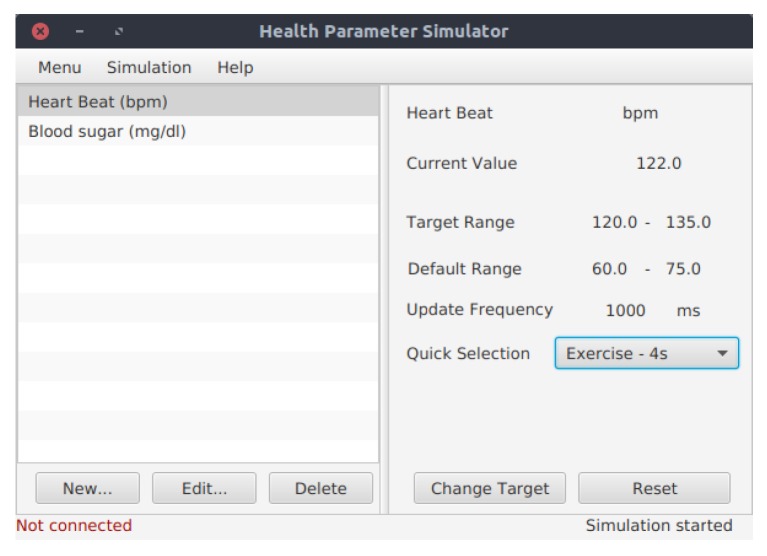
The simulator of health data.

E-care@home being a system combining health and environmental sensors, we need to record medical information in order to test the system and see their impact on the context and activity recognition. The Shimmer sensor described in Section 3.3 gathers various types of medical information (e.g., heart rate). However, it is impossible during a scenario to simulate the different problems we would like to monitor, such as a stress event, diabetes-related measures or stroke. It is also very improbable to record those events in non-simulated setups. To overcome this issue, we developed a health data simulator, presented in Figure 8, that sends health-related data to the database. This is completely transparent for the reasoner. Our current health simulator is very simplistic and requires the user to enter the target interval manually and will then select a random value in this interval. Work is currently done to improve the simulator and make it more realistic.

The E-care@home system has been tested using a scenario enabling the recognition of different activities depending on the context. This scenario is presented in Table 1. The second column in the table represents what the occupant of the home is doing, and the third column represents the activities recognized by the system. Most of the steps are self-explanatory, but Steps 8, 14 and 18 deserve some attention.

**Figure 9:**
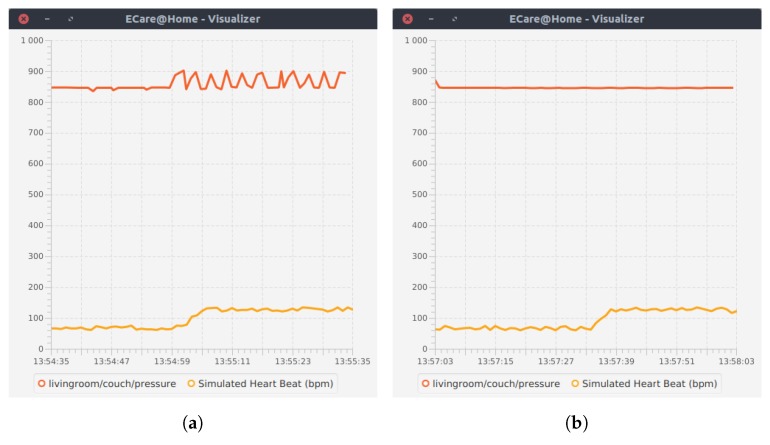

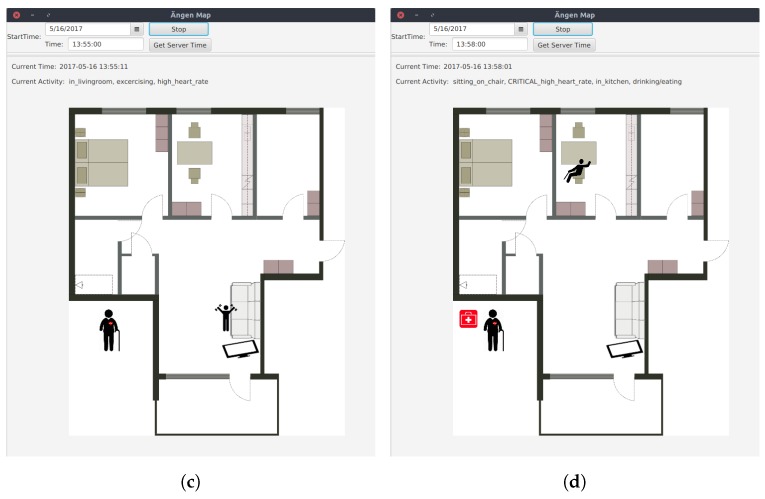
Time series signals from health and pressure sensor during the activities *exercising* and *stress event*. (**a**) The pressure and heart rate sensors during the exercising activity; (**b**) the pressure and heart rate sensors during the stress event; (**c**) recognized activities during the exercising activity; (**d**) recognized activities during the stress event.

In Step 8, the occupant is doing some physical exercises. In our scenario, the physical exercise consists of sitting down and getting up from the couch several times, usually during a minute. This produces a very specific pattern on the pressure sensor of the couch that can be recognized. During this time, the health data simulator is used to simulate an increased heart rate. In Step 18, the occupant experiences a stress event. Figure 9 shows the evolution of the pressure sensor and the heart beat during the activity *exercising* (Figure 9a) and during the stress event (Figure 9b), as well as the activities recognized (Figure 9c,d). It is important to note that the context here is essential to interpret the event *increased heart rate*. Indeed, in the case of exercising, the combination *specific pattern on pressure sensor + increased heart rate* enables the reasoner to infer the activity *exercising*, and this high heart rate is considered as “normal”. In the case of the stress event, the heart rate increases, and no other event can explain it. Therefore, the reasoner infers that this high heart rate is “critical”, meaning impossible to explain with the current data and might be a health emergency.

Step 14 shows another example of context recognition. The occupant started cooking (Step 11) and left the stove unattended (Step 14). After a certain amount of time, the reasoner will infer that the occupant forgot that he/she started to cook and that there is a fire hazard because the stove was left on. It will therefore create an event *burning*, and the system will be able to inform the user about the situation.

Each activity mentioned in the scenario is recognized based on preconditions, as explained in Section 5. A summary of the preconditions for our scenario is presented in Table 2.

### 6.1. Discussion about the Deployment

In this section, we discuss the deployment presented in the previous section and highlight some of the current advantages and limitations of the whole system. This section does not aim at giving a full performance evaluation for the reasoner; as such, an evaluation in terms of the performance of the reasoner in comparison with the off-the-shelf ontological reasoners can be found in previous publications [46]. The scalability of the proposed context recognition solution is still under study. A precise evaluation of such a system asks for longer observation processes with a higher number of sensors.

**Figure 10:**
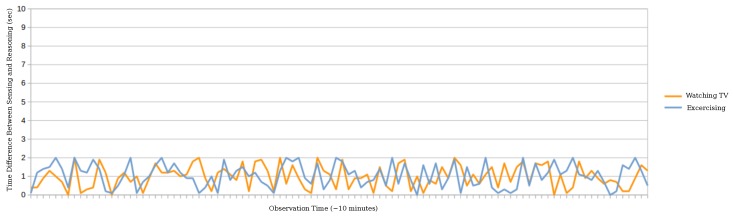
TimeDifference(Δt) between the sensing time and the reasoning time for two activities of *WatchingTV* and *Exercising*.

We have deployed the E-care@home system in a test apartment aimed to be a controlled environment. Currently, the system includes the least amount of sensors (seven sensor nodes) and 40 rules in order to recognize a total of 12 activities, each activity being associated with 3–5 rules. Assuming sensors are continuously capturing changes in the environment, the system always detects activities correctly according to the specified rules (which may be limited in scope as is the case with the *exercising* activity). The only concern is about the delay between the sensing and the inference time. With the given configuration, we have measured the performance of the context recognition process in terms of the time-difference (Δt) between the sensing time and the inference time for different activities. Figure 10 shows this relation for the two activities of *watchingTV* and *exercising*, both with more than one precondition. As we can see, the context recognition process monitoring the environment to capture the occurrence of these activities is almost acting in real time (less than 2 s). This trend to some extent reflects the scalability of the system and its reasonable degree of independence from the accumulation of sensor data grounded in the system, during a short observation process however (≈10 min).

The next step of the deployment is to install the system in real homes and test it in an unsupervised context for several days/weeks. In our controlled environment, some events and activities, in particular *exercising* or *burning*, have been simplified in order to be able to record them within the short period of time during which the tests took place and with the set of sensors that we considered. In a real configuration, it is obvious that such activities would be more complex and use different sensors and preconditions. In the case of exercising, for instance, we could install sensors on objects usually used for exercising, such as a mat or dumbbells. We could also provide the user with a wearable accelerometer and recognize move patterns related to exercising. Similarly, the event *burning* could be recognized with a combination of smoke and temperature sensors installed near the stove. From the system’s point of view, such changes would imply a reconfiguration of the rules to detect these events.

Currently, the system relies only on hard-coded rules represented in the ontology (see Figure 4 as an example) to infer context and activities. This solution enables easy reconfiguration for new homes and does not require any data to train. However, it implies that the system performance is directly linked to the precision of the rules’ definition. If the rules are not well-defined, the system will not be able to perform accurate recognition. Hybrid approaches combining data-driven and model-driven techniques could be considered in the future.

As do most of the other context-recognition systems, E-care@home considers a single occupant in the home. In real situations, however, users would receive guests and might even not live alone. Work is currently being done to be able to count the number of occupants in a smart home with simple sensors in order to improve the reasoning. Preliminary results on this problem can be found in [47].

As mentioned above, the next step is to configure the test apartment for more realistic scenarios with a longer observation process. New challenges arise with this step. Those challenges include, but are not limited to: annotations and ground truth to evaluate the performance of the system, long-term robustness, scalability of the reasoner and multi-occupant scenarios.

## 7. Towards Automatic Network Configuration: Configuration Planning

So far, our focus has been placed on the use of the framework for activity recognition. However, here, we would like to also present suggestions for the same framework to be used for the automatic configuration of devices. In the ambient assisted living context, it is important that users can express their queries in terms of activities and locations without having to refer to specific instances of sensors or actuators. In addition, there may be multiple ways to infer the same information, and available sensors and actuators can vary over time and be different from one home to another. This leads to a need for some form of decision making regarding which sensors are used in what way to achieve information goals. We propose that the problem of automation configuration is seen as a planning problem where configuration planning [48,49,50,51] aims at automatically finding a set of sensor and actuator instances to use for gathering and processing data and to answer information queries.

Configuration planning has similarities to web service composition [52], which also concerns flows of information and where task planning techniques often are employed. We recently proposed and compared two solutions to temporal configuration planning [51] in which we deal not only with information dependencies, but also with the temporal intervals during which information is available. This allows us, for instance, to reason about resources (such as robots) that may be occupied while they provide required information.

We plan to extend this approach by integrating it with the E-care@home ontology. This will allow us to generate configuration planning domains based on knowledge contained in the ontology and, thus, reducing the amount of knowledge engineering required when setting up a new smart environment. Ideally, it should suffice to specify which devices and services are provided in the new environment in the a-boxof the ontology. With this, the ontology can be used to derive sensing, configuration and information capabilities and automatically generate a domain for task and configuration planning. If, for instance, we would specify that a light sensor is attached to the LED of a stove, the ontology could tell us that this allows us to determine if the stove is on or off. Stove usage could then be combined with information about a person’s whereabouts to determine if that person is cooking. Removing the need to specify all of these relations by hand and inferring it from an ontology would allow significantly easier setup of new environments. In addition, this also reduces knowledge redundancy between the domain definition of the planner and the knowledge in the ontology.

Network scheduling is used for ensuring that commands coming from the configuration planner are satisfied at the low level of operation. Here, one aspect that is highly important for E-care@home is the retrieval of data within fixed time limits. The scheduled protocol TSCH is used for configuring and producing the schedules that play a crucial role in the resulting operation of the network. We plan to study and experimentally evaluate the trade-offs of possible schedules in reliability, latency, throughput and energy, as well as possible benefits of allowing the configuration planner to influence low-level sensor network communication schedules.

## 8. Conclusions

In this paper, we have presented a system, E-care@home, that is able to augment devices and their measurement into semantic representation upon which further information can be extracted, e.g., in terms of activities in the home. A modular approach to model information using ontologies was used, and the potential for this approach to be used for other scenarios such automatic configuration was discussed. An experimental scenario conducted in a smart home environment has been described illustrating the ability of the system to recognize activities. It was demonstrated that the ontology has the capacity to represent data at varying levels of abstraction and to provide an adequate foundation upon which a non-monotonic reasoner can deduce activities. Further, we have shown that the reasoning time is not influenced as the volume of data increases. Future work will focus on extending the context recognition system and its evaluation with more realistic scenarios with a larger number of users and more complex activities. There is an increasing trend to use smart environments in order to promote independent living for people in their homes. Today, many commercial systems are available that provide the basic sensor infrastructure, but lack the ability to give a rich context awareness. Rather, such systems simply can alarm in the case of very simple sensor events.

## Figures and Tables

**Table 1:** Example of a single occupant scenario.

	Step	Activities Recognized
1	Occupant 1 turns the TV on and sits on the couch	in living room, sitting, watching TV
2	Occupant 1 gets up and fetch the TV program on the table	in living room, moving, watching TV
3	Occupant 1 sits down and watch TV	in living room, sitting, watching TV
4	Occupant 1 goes to the bathroom	in living room, moving
5	Occupant 1 is in the bathroom	in bathroom, moving
6	Occupant 1 goes to the living room	in living room, moving, watching TV
7	Occupant 1 turns the TV off	in living room, moving
8	Occupant 1 exercises	in living room, exercising, high heart rate
9	Occupant 1 goes to the kitchen	in living room, moving
10	Occupant 1 goes to the kitchen	in kitchen, moving
11	Occupant 1 turns the oven on and starts cooking	in kitchen, moving, cooking
12	Occupant 1 goes to the living room	in kitchen, moving, cooking
13	Occupant 1 goes to the living room	in living room, moving, cooking
14	Occupant 1 sits down and read a book	in living room, sitting, burning
15	Occupant 1 goes to kitchen	in living room, moving, cooking
16	Occupant 1 goes to kitchen	in kitchen, moving, cooking
17	Occupant 1 turns the oven off	in kitchen, moving
18	Occupant 1 sits on a chair and drinks tea (stress event)	in kitchen, eating, critical high heart rate

**Table 2:** Activities detected, their preconditions and the sensors used during the detection.

Activity Recognized	Preconditions	Sensors Used
in living room	motion for 3 s or more in the living room	motion sensor
in kitchen	motion for 3 s or more in the kitchen	motion sensor
in bathroom	motion for 3 s or more in the bathroom	motion sensor
sitting	pressure on the couch or the chair	pressure sensor
moving	motion for 3 s or more on any motion sensor	motion sensor
watching TV	TV turned on and occupant in the same room	motion sensor + TV luminosity sensor
changing pose	standing up or sitting down	pressure sensor
exercising	at least 4 times changing pose within 8 s	pressure sensor
cooking	oven turned on	oven luminosity sensor + motion sensor in the kitchen
burning	cooking lasts for more than 50 s	oven luminosity sensor
eating	cooking ends recently (within recent 10 s)	pressure sensor
stressing	no exercising process detected within recent 10 s	heart beat simulator
